# Bushen Huatan Formula Ameliorates Early Chronic Low‐Grade Inflammation in Obese PCOS Via the TLR4/NF‐κB Signaling Pathway: An Integrated Network Pharmacology and Experimental Study

**DOI:** 10.1002/iid3.70518

**Published:** 2026-09-14

**Authors:** Jianing Zhang, Fangyun Gu, Yunyun Li, Xiaoling Feng, Hongying Kuang, Fang Xu, Miao Sun

**Affiliations:** ^1^ HeilongJiang University of Chinese Medicine Harbin China; ^2^ Department of Gynecology Shenzhen Qianhai Shekou Free Trade Zone Hospital Shenzhen China; ^3^ The First Affiliated Hospital of Heilongjiang University of Chinese Medicine Harbin China

**Keywords:** BuShenHuaTan formula, Chinese medicine, chronic low‐grade inflammation, network pharmacology, polycystic ovary syndrome

## Abstract

**Objective:**

This study used an integrated network pharmacology and experimental strategy to investigate how Bushen Huatan Formula (BHF) mitigates early‐stage chronic low‐grade inflammation in obese polycystic ovary syndrome (PCOS).

**Methods:**

Candidate components and targets of BHF were retrieved from the TCMSP database, and protein–protein interaction, GO and KEGG enrichment, and molecular docking analyzes were performed. Predictions were then tested in a letrozole‐induced PCOS rat model, in which ovarian and adipose histopathology and TLR4/NF‐κB expression were examined at 6 and 12 weeks; isolated granulosa cells (GCs) were further exposed to BHF‐containing serum to quantify inflammatory cytokines.

**Results:**

Ten key components and 169 intersecting targets were obtained, with enrichment in the NF‐κB, IL‐17, and MAPK pathways. Docking indicated favorable binding (< –7.0 kcal/mol) of core components to targets such as AKT1 and TNF. In model rats, inflammation increased in a time‐dependent manner, accompanied by elevated TLR4 and NF‐κB in both adipose and ovarian tissues, whereas BHF‐containing serum suppressed TNF‐α, IL‐1β, IL‐6, and IL‐8 in GCs. This effect was more pronounced at the early stage (6 weeks) than at the later stage (12 weeks).

**Conclusion:**

BHF may attenuate early chronic low‐grade inflammation in PCOS, likely through modulation of the TLR4/NF‐κB axis, supporting its potential value for early intervention.

## 1 Introduction

Polycystic ovary syndrome(PCOS)is a common endocrine disorder affecting approximately 20% of women of reproductive age worldwide [1]. Its major clinical features include chronic anovulation, hyperandrogenism, and polycystic ovarian morphology [2]. Recent studies have revealed that patients with PCOS commonly exhibit chronic low‐grade inflammation(CLGI), characterized by significantly elevated levels of serum inflammatory cytokines [3]. This inflammatory state is closely associated with the core pathophysiological alterations of PCOS [3, 4]. CLGI in PCOS is not only evident in the systemic circulation but is particularly pronounced in the ovarian microenvironment, especially within Granulosa Cells (GCs) [5].

Granulosa cells, which surround oocytes within the follicles, play a crucial role in folliculogenesis, and inflammatory cytokines regulate their functions. Pathological alterations in GCs have been identified as contributing factors in PCOS and other ovarian disorders such as premature ovarian failure [6]. Recent studies [7]have shown that inflammatory cytokines, including IL‐17 (Interleukin‐17), IL‐8, IL‐1β, IL‐18, and TNF‐α (Tumor Necrosis Factor‐α), are significantly elevated in the follicular fluid of PCOS patients, accompanied by increased GC apoptosis. Furthermore, the ovarian inflammatory microenvironment may lead to abnormal follicular development and ovulatory dysfunction [8]. Such aberrant cytokine secretion, via paracrine and autocrine mechanisms, further disrupts normal GC physiological functions, exerting multiple effects on the pathophysiology of PCOS.

In recent years, the therapeutic efficacy of various traditional Chinese medicine (TCM) formulations in the treatment of PCOS has gained increasing recognition. According to TCM theory, the fundamental pathogenesis of PCOS is kidney deficiency and phlegm‐dampness stagnation. Extensive studies have confirmed that TCM approaches such as qi‐tonifying, phlegm‐resolving, and blood‐activating therapies exhibit significant effects on chronic low‐grade inflammation, and the advantages of TCM in treating PCOS are increasingly recognized [9, 10, 11]. BuShenHuaTan Formula(BHF), a clinically effective TCM prescription, consists of five herbal components: Huang Qi (HQ), Yin Yang Huo (YYH), Cang Zhu (CZ), Fu Ling (FL), and Dan Shen (DS). Clinical studies have demonstrated that BHF effectively alleviates insulin resistance and improves ovarian ovulatory function in patients with PCOS [12]. In a previous study, we applied liquid chromatography–mass spectrometry (LC‐MS)‐based metabolomics to investigate the therapeutic effects of BHF in PCOS patients, revealing that improvements in phospholipid metabolism and alterations in amino acid metabolism played key roles [13]. The results suggested that BHF exerts therapeutic effects in PCOS patients by mitigating inflammatory responses and oxidative stress. Collectively, preliminary studies indicate that BHF possesses unique advantages in treating chronic low‐grade inflammation in PCOS and may exert multi‐target therapeutic effects, although the precise mechanisms remain to be elucidated.

Given the complex pathophysiological characteristics of PCOS and the multi‐component, multi‐target nature of TCM formulations, traditional single‐target research approaches are insufficient to comprehensively elucidate the mechanisms of BHF. Therefore, this study employed network pharmacology by integrating TCM pharmacology databases (TCMSP), protein–protein interaction (PPI) network analysis, functional enrichment analysis, and molecular docking to systematically investigate the active components, potential targets, and signaling pathways of BHF. Moreover, based on previous clinical and animal model studies of PCOS [13, 14], letrozole (LET)‐induced PCOS rat models exhibit reproductive and metabolic abnormalities similar to those observed in clinical PCOS patients. To this end, in order to validate the reliability of the network pharmacology predictions, this study established a LET‐induced PCOS rat model. This model, combined with experimental verification, was utilized to investigate the regulatory mechanisms underlying the inflammatory response in PCOS and to elucidate the potential mechanism of action of BHF in the early‐stage low‐grade inflammation associated with PCOS.

Although previous studies have reported the involvement of the TLR4/NF‐κB pathway in the pathogenesis of PCOS and the anti‐inflammatory effects of certain traditional Chinese medicine (TCM) formulations, the specific role of Bushen Huatan Formula (BHF) in regulating the chronic low‐grade inflammatory microenvironment of PCOS—particularly its dynamic evolution during disease progression—remains poorly defined. The present study therefore integrates network pharmacology, molecular docking and multi‐level experimental validation to examine how BHF modulates early‐stage inflammation in PCOS. Relative to earlier work on TCM and the TLR4/NF‐κB axis, the contribution of this study is threefold: the ovarian inflammatory microenvironment is characterized at two defined stages of disease (6 and 12 weeks) rather than at a single endpoint, so that the stage‐dependent efficacy of the intervention can be assessed; the analysis is directed at the obese PCOS phenotype, in which chronic low‐grade inflammation is most pronounced; and a complete multi‐herb formula, rather than a single herb or an isolated compound, is evaluated at both the systems and the cellular level. This was addressed through the following steps: ①Screening active drug components and potential targets based on the TCMSP database; ②Investigating drug–disease pathways through PPI network and enrichment analyzes to identify core active components and key target proteins; ③Evaluating the binding properties of core active components with key proteins via molecular docking to provide molecular‐level theoretical support; ④Establishment of a PCOS rat model to validate the core inflammatory signaling pathway and comparing the inflammatory status at two disease stages (6 and 12 weeks) to capture the dynamic progression of inflammation and the stage‐dependent efficacy of BHF. This study aims to systematically elucidate the early inflammatory regulatory mechanisms in PCOS, as well as the modulatory role of the Bushen Huatan Formula (BHF), through both network pharmacology and animal experiments. It further seeks to clarify the functional network through which BHF regulates the PCOS inflammatory microenvironment. The findings are intended to provide robust experimental data and a theoretical foundation for the modern clinical research of Traditional Chinese Medicine in treating PCOS.

## 2 Materials and Methods

### 2.1 Network Pharmacology Study

#### 2.1.1 Screening of BHF Chemical Components and Targets

The chemical components of the five herbs in BHF (Huang Qi, Yin Yang Huo, Cang Zhu, Fu Ling, and Dan Shen) were retrieved from the TCMSP database (https://www.tcmsp-e.com/index.php). Active components were screened based on oral bioavailability (OB) ≥ 30% and drug‐likeness (DL) ≥ 0.18. Additionally, potential targets of the active components were retrieved from the TCMSP database. To ensure data standardization and adherence to scientific norms, all targets were standardized using the UniProt protein database (https://www.uniprot.org/) with species limited to Homo sapiens.

#### 2.1.2 Collection of PCOS Disease Targets

Relevant disease targets were searched in the GeneCards (https://www.genecards.org) and OMIM (http://www.omim.org) databases using the keywords “PCOS” and “polycystic ovary syndrome.” In the GeneCards database, each target is associated with a Score. Because PCOS is a heterogeneous, multifactorial endocrine and metabolic disorder whose pathogenic target network has not yet been fully delineated, a deliberately inclusive threshold (relevance score ≥ 1) was applied to retain as many potentially disease‐relevant targets as possible and to avoid the premature exclusion of biologically meaningful candidates. The specificity of the ultimately identified core targets was safeguarded by the subsequent stepwise screening procedure, comprising intersection with the predicted BHF component targets, topological ranking within the PPI network, and functional enrichment analysis. After standardizing gene names via the UniProt database, redundant entries were removed to obtain the final list of PCOS therapeutic targets. The intersection of BHF and PCOS targets was analyzed online using the Venny 2.1.0 platform (https://bioinfogp.cnb.csic.es/tools/venny/index.html), and a Venn diagram of drug–disease targets was generated.

#### 2.1.3 Construction of the “Herb–Component–Target” Network

A visual “Herb–Component–Target” network was constructed using Cytoscape v3.10.3, where nodes represent herbs, chemical components, or targets, and edges represent the relationships among them. Network topology was analyzed using the built‐in Network Analyzer module, and the degree of each node was quantitatively calculated as a key parameter. In topological terms, the degree of a node denotes the number of edges directly connecting it to other nodes; components with higher degree values interact with a greater number of targets and are therefore generally regarded as occupying more central and influential positions within the network. Accordingly, the components were ranked in descending order of degree, and the top 10 were selected as the core active ingredients of BHF.

#### 2.1.4 PPI Network Analysis and Core Target Screening for BHF in PCOS

After integrating and analyzing the network of active herbal components and potential PCOS targets, the intersecting targets were imported into the STRING database (https://www.string-db.org/) with the analysis mode set to “Multiple proteins,” species limited to Homo sapiens, and minimum required interaction score set to “medium confidence (> 0.4),” with other settings left at default, to construct the Protein‐Protein Interaction(PPI) network. The network was imported into Cytoscape v3.10.3, where topology was analyzed using the built‐in Network Analyzer module. Each target was ranked according to its degree value, and the top 10 targets were identified as core targets using the CytoHubba plugin. A hierarchical PPI network was then visualized.

#### 2.1.5 GO and KEGG Pathway Enrichment Analysis

Enrichment analysis of the intersecting targets was performed using the Metascape platform (https://metascape.org/). The intersecting targets were input into Metascape with the species set to H. sapiens and parameters: Min Overlap = 3, *p* value cutoff = 0.01, and Min Enrichment = 1.5. Enrichment analyzes were conducted based on Gene Ontology (GO) annotations, including Biological Processes (BP), Molecular Functions (MF), and Cellular Components (CC), as well as Kyoto Encyclopedia of Genes and Genomes (KEGG) pathways. Visualization and graphical enhancement were performed using the bioinformatics platform (https://www.bioinformatics.com.cn/) to clearly present key biological processes and signaling pathways.

#### 2.1.6 Molecular Docking

Molecular docking was performed using AutoDock Vina v1.1.2 to validate the interactions between the top 10 BHF components (based on degree) and the top 10 core targets from PPI network analysis, obtaining docking binding energies and conformations. The specific procedures were as follows: ① Preparation of small molecule ligands: corresponding mol2 files were downloaded from TCMSP and minimized using Chem3D v23.1.1, then saved in a standardized format; ②Preparation of protein receptors: the top 10 core targets were retrieved from UniProt with “Reviewed (Swiss‐Prot)” and “Human” filters, and corresponding PDB files of protein structures were downloaded from RCSB PDB (https://www.rcsb.org/) and processed using PyMOL Anaconda3 to remove water molecules and small‐molecule ligands; ③Hydrogens were added to receptor proteins using AutoDockTools v1.5.7 and docking boxes were generated based on protein structures; ④Docking of prepared ligands and protein receptors was conducted using AutoDock Vina v1.1.2, and the resulting binding energies were exported and organized. Binding energies < −7.0 kcal/mol were considered stable; ⑤Molecular docking results were visualized using PyMOL Anaconda3.

### 2.2 Experimental Materials and Preparation

#### 2.2.1 Animals

Female Wistar rats (Specific Pathogen Free grade, weighing 150–200 g) were purchased from the Animal Laboratory of HeilongJiang University of Chinese Medicine (Harbin, China). All rats were housed in individually ventilated cages under controlled conditions (temperature 21°C–22°C, humidity 55%–65%, 12/12‐h light‐dark cycle) with ad libitum access to food and water. All animal experiments were performed in accordance with the Guide for the Care and Use of Laboratory Animals and with the Regulations for the Administration of Affairs Concerning Experimental Animals of the People's Republic of China, and the protocol was reviewed and approved by the Animal Ethics Committee of Heilongjiang University of Chinese Medicine. The animal work described here was carried out in 2018. At that time the institutional review procedure did not issue serially numbered approval certificates, and the Animal Ethics Committee of the First Affiliated Hospital of Heilongjiang University of Chinese Medicine, which now assigns such numbers, was not established until after 2020. No approval number is therefore available for this study.

At 21 days of age, the female Wistar rats were randomly divided into four groups: Group Con 1 (*N* = 20, 200 μg/day; continuous administration of placebo pellets for 60 days), Group LET 1 (*N* = 20, 200 μg/day; continuous administration of LET pellets for 60 days), Group Con 2 (*N* = 20, 200 μg/day; continuous administration of placebo pellets for 90 days), and Group LET 2 (*N* = 20, 200 μg/day; continuous administration of LET pellets for 90 days). LET (letrozole) was purchased from MedChemExpress. The 60‐day and 90‐day continuous‐release LET pellets were customized by Innovative Research of America, and the corresponding placebo pellets were manufactured by the same company. The dosage of 200 μg/day was selected based on previous studies [15]. Starting from the second week post‐implantation, food intake, body weight, and body temperature were measured daily and calculated weekly. Rats in Group LET 1 and Group Con 1 were euthanized and tissues were harvested at 6 weeks of age (post‐implantation), while those in Group LET 2 and Group Con 2 were euthanized and tissues were harvested at 12 weeks of age (Supporting Information Table S3). The 6 and 12 week time points were selected to represent an early, developing stage and a fully established stage of the letrozole‐induced PCOS model, respectively [14], enabling the dynamic progression of the inflammatory microenvironment and the stage‐dependent efficacy of BHF to be assessed.

#### 2.2.2 Preparation and Administration of BHF

BHF consists of five herbal medicines, and the information and dosages of the components are shown in Table 1. All five herbs were provided by the Chinese Herbal Medicine Department of the First Affiliated Hospital of HeilongJiang University of Chinese Medicine (Harbin, China). The herbs were weighed, cleaned, and decocted in 800 mL of distilled water for 30 min. After two rounds of decoction, the extracts were combined. The combined decoction was aliquoted into soft storage bags, vacuum‐sealed, and stored at 5°C–10°C until use.

**Table 1:** The composition of BHF.

Pinyin name	Latin name	English name	Content (g)
Huang Qi	*Astragalus membranaceus*	Membranous milk vetch root	30
Yin Yang Huo	*Herba epimedii*	Epimedium herb	20
Cang Zhu	*Rhizoma atractylodis*	The rhizome of Chinese atractylode	15
Fu Ling	*Poria cocos*	Tuckahoe	15
Dan Shen	*Salvia miltiorrhiza*	The root of red‐rooted salvia	10

#### 2.2.3 Preparation of BHF‐Containing Serum

A Total of 10 6–7‐week‐old female Wistar rats (150–200 g) were acclimated to the environment with free access to food and water for 7 days. Rats in the BHF‐serum group (*N* = 5) were orally administered the BHF decoction for 3 consecutive days at a dose of 1 mL/100 g twice daily at 09:00 and 18:00. Rats in the control group (*N* = 5) received the same volume of physiological saline at the same time points. 30 min after the last administration, rats were intraperitoneally injected with sodium pentobarbital (0.2 mL/100 g), and approximately 6 mL of abdominal aortic blood was collected. Blood samples were incubated in a 56°C water bath for 30 min, centrifuged at 4000 rpm for 15 min, filtered through a 0.22 μm filter, and transferred to new Eppendorf tubes for storage at −80°C for subsequent cellular experiments [16]. A serum concentration of 15% was used in all subsequent experiments; this lies within the 10%–20% range conventionally applied in serum pharmacology studies of Chinese herbal formulae and was selected on the basis of preliminary optimization experiments performed in our laboratory.

### 2.3 Experimental Methods and Procedures

#### 2.3.1 Fasting Blood Glucose Assays

Massage the tail vein of overnight fasted rats and disinfect it with 75% ethanol. After the skin is dry, take 1 ml of blood. Pay attention to make the blood flow out naturally and avoid extrusion. Blood samples were centrifuged (3000 rpm, 10 min, 4°C) (Remi, C‐24 BL, Mumbai, India). Glucose in serum was measured by Glucose Oxidase and Peroxidase (GOD‐POD kit) kit according to the manufacturer's instructions.

#### 2.3.2 H&E Staining and Immunohistochemistry

Rats from Group LET 1 and Group LET 2 were euthanized for tissue collection at 6 and 12 weeks of LET administration, respectively (*N* = 10). Ovaries and inguinal fat were collected freshly and weighed. One ovary and 0.5 cm length part of inguinal fat were used for histology. To observe ovary and fat morphology changes, hematoxylin and eosin (H&E) staining was used. Ovary and inguinal fat pads were fixed in 10% neutral buffered formalin for 24 h, and stored in 70% ethanol before performing H&E staining and immunohistochemistry. For H&E staining, tissues were embedded in paraffin using standard techniques.

Ovaries and inguinal fat pads were respectively, serially sectioned at 5 μm and then stained with hematoxylin and eosin. Images were collected using Olympus DP73 microscope. For immunohistochemistry, the protocol was performed as previously described [17]. Briefly, the ovary and inguinal fat sections were dewaxed and rehydrated, and antigen retrieval was performed in 0.1 M sodium citrate buffer for 10 min in a microwave oven. Sections were incubated in 3% hydrogen peroxide for 15 min to eliminate endogenous peroxidases. Sections were blocked with goat serum for 15 min at 37°C followed by incubation overnight at 4°C with primary antibodies Toll‐like receptor 4 (TLR4) (1: 200, WL00196, wanleibio, China) and nuclear factor‐κB (NF‐κB) (1: 200, WL01980, wanleibio, China). Following three washes in PBS, sections were incubated with biotin‐labeled goat anti‐rabbit IgG (H + L) (1: 500, #31460, ThermoFisher, USA) for 30 min at 37°C. Signals were visualized by reaction with the DAB peroxidase substrate kit (DA1010, Solarbio, China). Images were acquired using Olympus DP73 microscope. As negative controls, sections were incubated with diluent at the absence of primary antibody.

#### 2.3.3 qRT‐PCR Array Analysis

92 related gene expression profiles were simultaneously analyzed using rat NF‐κB Signaling Targets qPCR Array (Supporting Information Table S1) according to the manufacturer's protocol (Wcgene Biotech, Shanghai, China). Array data was validated using real time quantitative reverse transcription PCR (qRT‐PCR). Total RNA (1 μg) was isolated using Trizol reagent and subjected to reverse transcription reactions with random hexamer primers using the High Capacity cDNA Reverse Transcription kit (Applied Biosystems, Foster City, CA). qRT‐PCR was performed on a 7900HT Sequence Detection system (Applied Biosystems) using FastStart Universal SYBR Green Master kit (Roche Diagnostics, Indianapolis, IN) and TaqMan Universal Master Mix (Applied Biosystems) following the manufacturer's instructions. GAPDH was used as a housekeeping control gene. Each test was done in triple replication and the 2^−ΔΔCt^ method was used to calculate the expressions of genes. Data were analyzed using Wcgene Biotech software. Genes with fold‐changes more than or less than 2.0 were considered to be of biological significance.

#### 2.3.4 Isolation and Culture of Granulosa Cells From LET‐Induced PCOS Rats

Following 6 weeks of pellet administration, rats from Group Con 1 and Group LET 1 were euthanized for ovarian excision. Similarly, rats from Group Con 2 and Group LET 2 were euthanized after 12 weeks of pellet administration for ovarian excision (*N* = 10). To isolate granulosa cells (GCs), the ovaries were immersed in culture medium (DMEM containing 5% FBS) and punctured with a 26‐gauge needle to release GCs [18]. The GCs were then seeded into 3.5 cm culture dishes and cultured under standard conditions (DMEM containing 15% FBS, 37°C, 5% CO_2_). After 48 h of culture, a portion of GCs isolated from Group Con 1, Group LET 1, Group Con 2, and Group LET 2 were transferred and subsequently cultured for an additional 48 h in either DMEM containing 15% control serum or DMEM containing 15% BHF serum for subsequent assays.

#### 2.3.5 ELISA

Cell supernatants were collected after centrifugation, and ELISA kits (Bioswamp, China) were used to measure TNF‐α (WLE05c, Wanlei Bio, China), IL‐1β (WLE03c, Wanlei Bio, China), IL‐6 (WLE04c, Wanlei Bio, China), IL‐8 (DG20725D, Doges, China), LBP (SEB406Ra, Uers, China), LPS (IEB526Ge, Uers, China), CD14 (SEA685Ra, Uers, China), and hsCRP (JL10287, Jianglai Bio, China) levels. All measurements were performed strictly according to the manufacturer's instructions.

**Figure 1:**
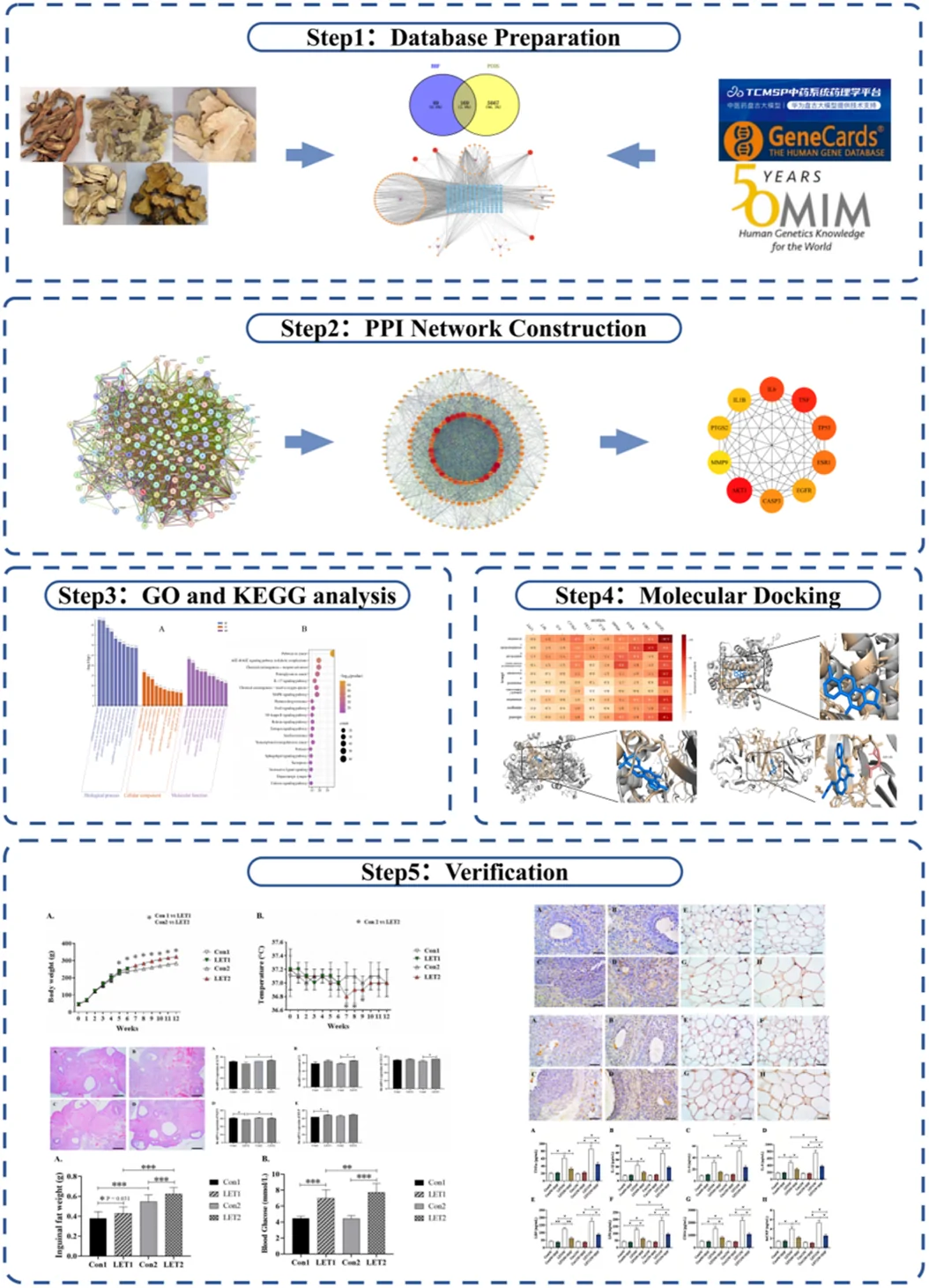
Diagram of the experimental procedure.

A schematic diagram of the experimental protocol is presented in Figure 1 (Created with BioGDP.com).

### 2.4 Statistical Analysis

Statistical analyzes were performed using SPSS v21.0 (SPSS, Chicago, IL, USA) and GraphPad Prism 8.00 (GraphPad Software, La Jolla, CA, USA). In in vitro studies, statistical significance among groups was determined using one‐way ANOVA followed by Dunnett's post hoc test. Data are presented as mean ± standard error of the mean (S.E.M.), and *p* < 0.05 was considered statistically significant.

## 3 Results

### 3.1 Network Pharmacology Study

#### 3.1.1 Screening of BHF Chemical Components and Targets

**Figure 2:**
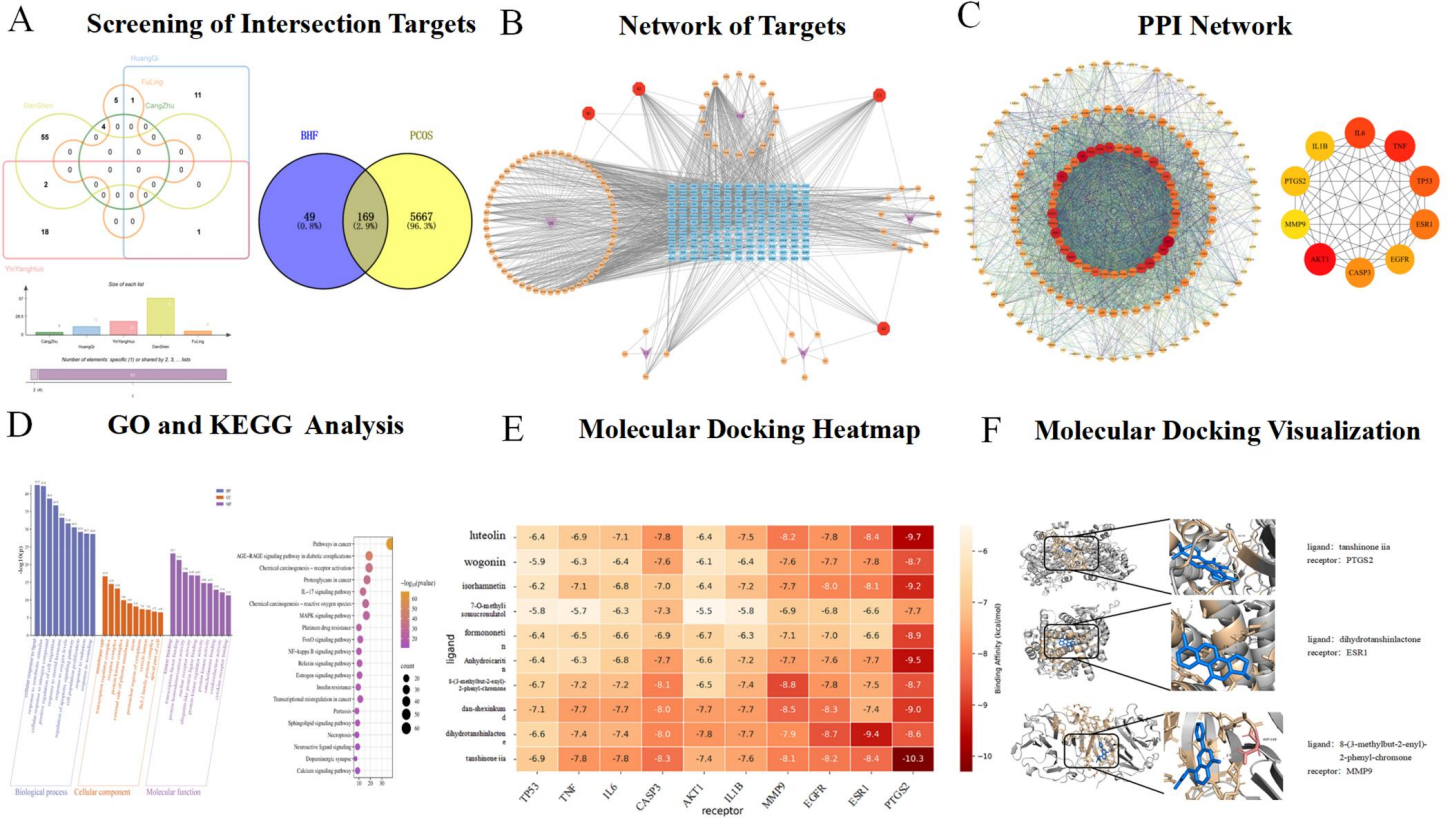
Results of network pharmacology analysis.

According to the screening criteria, 101 chemical components were retrieved from the TCMSP database, including 13 from Huang Qi, 21 from Yin Yang Huo, 57 from Dan Shen, 6 from Fu Ling, and 4 from Cang Zhu. Among these 101 active components, one was shared by Fu Ling and Huang Qi, two were shared by Yin Yang Huo and Dan Shen, and one was shared by Huang Qi and Yin Yang Huo (Figure 2A). Targets of these 101 chemical components were predicted using the TCMSP database. After standardizing the target names via the UniProt database and removing duplicates, a total of 218 chemical component targets for BHF were obtained.

#### 3.1.2 Screening of PCOS‐Related Targets

Disease targets were collected from the GeneCards and OMIM databases using the keywords “PCOS” and “polycystic ovary syndrome.” The collected disease targets were merged and deduplicated, resulting in a total of 5836 disease targets. Online analysis via the Venny website identified 169 intersecting targets between the 218 drug targets and 5836 disease targets, which were visualized in a Venn diagram (Figure 2A).

#### 3.1.3 Construction of “Herb–Component–Target” Network

Based on the selected intersecting targets, a BHF regulatory network was constructed using Cytoscape v3.10.3 (Figure 2B), analyzing interactions among herbs, components, and disease targets. Purple arrows indicate the five herbs of BHF, surrounding circles represent the corresponding active chemical components of each herb, red octagons represent shared chemical components among the herbs, and blue rectangles represent the intersecting targets between BHF and PCOS. Components were ranked according to their “Degree” values, and the top 10 herbal components were selected as the key active ingredients of BHF (Table 2), potentially representing bioactive compounds with therapeutic potential against chronic low‐grade inflammation in PCOS.

**Table 2:** Top 10 active compounds of BHF for the treatment of PCOS with degree value.

Name	Degree
Luteolin	116
Wogonin	34
Tanshinone iia	30
Formononetin	27
Isorhamnetin	26
7‐O‐methylisomucronulatol	26
Anhydroicaritin	26
Dan‐shexinkum d	23
Dihydrotanshinlactone	22
8‐(3‐methylbut−2‐enyl)‐2‐phenyl‐chromone	22

#### 3.1.4 Establishment of PPI Network and Core Target Screening for BHF Treatment of PCOS

The intersecting targets were imported into the STRING database with the analysis mode set to “Multiple proteins,” species limited to Homo sapiens, and minimum required interaction score set at “medium confidence (> 0.4)”; other settings were default. The resulting PPI network (Figure 2C) was imported into Cytoscape v3.10.3, where targets were ranked by Degree value. The top 10 targets were identified as core targets (Table 3): AKT1, TNF, IL6, TP53, ESR1, CASP3, EGFR, IL1B, PTGS2, and MMP9 (Figure 2C).

**Table 3:** Top 10 protein of BHF for the treatment of PCOS with degree value.

Name	Degree
AKT1	124
TNF	119
IL‐6	116
TP53	114
ESR1	110
CASP3	109
EGFR	108
IL1B	107
PTGS2	107
MMP9	106

#### 3.1.5 GO and KEGG Pathway Enrichment Analysis

GO and KEGG enrichment analyzes of the potential therapeutic targets were performed using the Metascape website. Results indicated that BHF targets in PCOS are involved in multiple therapeutic pathways. GO enrichment analysis (Figure 2D) showed that major biological processes (BP) involved Cellular response to lipid, Response to steroid hormone, and Regulation of apoptotic signaling pathway; main cellular components (CC) included Membrane raft, Transcription regulator complex, and Receptor complex; key molecular functions (MF) included Kinase binding, Transcription factor binding, and Oxidoreductase activity. KEGG pathway enrichment analysis (Figure 2D) identified the top 20 significantly enriched pathways associated with BHF and PCOS (Table 4), including the AGE‐RAGE signaling pathway in diabetic complication, IL‐17 signaling pathway, MAPK signaling pathway, NF‐κB signaling pathway, Estrogen signaling pathway, and Insulin resistance.

**Table 4:** KEGG pathway enrichment analysis.

Pathway	P	Count
Pathways in cancer	7.24 × 10^−68^	64
AGE‐RAGE signaling pathway in diabetic complications	5.62 × 10^−48^	32
Chemical carcinogenesis ‐ receptor activation	3.24 × 10^−36^	32
Proteoglycans in cancer	2.24 × 10^−32^	29
IL‐17 signaling pathway	1.51 × 10^−31^	23
Chemical carcinogenesis ‐ reactive oxygen species	5.13 × 10^−28^	27
MAPK signaling pathway	4.79 × 10^−26^	28
Platinum drug resistance	5.01 × 10^−23^	17
FoxO signaling pathway	1.55 × 10^−21^	19
NF‐kappa B signaling pathway	2.40 × 10^−20^	17
Relaxin signaling pathway	3.31 × 10^−20^	18
Estrogen signaling pathway	1.17 × 10^−19^	18
Insulin resistance	1.66 × 10^−18^	16
Transcriptional misregulation in cancer	4.68 × 10^−18^	19
Pertussis	1.26 × 10^−17^	14
Sphingolipid signaling pathway	3.16 × 10^−16^	15
Necroptosis	3.47 × 10^−13^	14
Neuroactive ligand signaling	4.79 × 10^−13^	15
Dopaminergic synapse	1.23 × 10^−11^	12
Calcium signaling pathway	1.62 × 10^−11^	15

#### 3.1.6 Molecular Docking Results

Molecular docking analyzes were performed for 10 key BHF components against 10 core targets (AKT1, TNF, IL6, TP53, ESR1, CASP3, EGFR, IL1B, PTGS2, MMP9), and the results were compiled and visualized (Figure 2E). Among the 100 molecular docking interactions between the 10 herbal components and 10 core targets, 66 demonstrated strong binding affinity (< −7.0 kcal/mol), indicating stable ligand–receptor interactions. Representative ligands and receptors with the lowest binding energies were visualized (Figure 2F). Notably, PTGS2 exhibited the strongest binding affinity with tanshinone IIA, highlighting its potential as a lead compound in BHF for treating chronic low‐grade inflammation in PCOS.

### 3.2 Cellular Experiments

#### 3.2.1 Body Weight Accumulation and Temperature Changes

**Figure 3:**
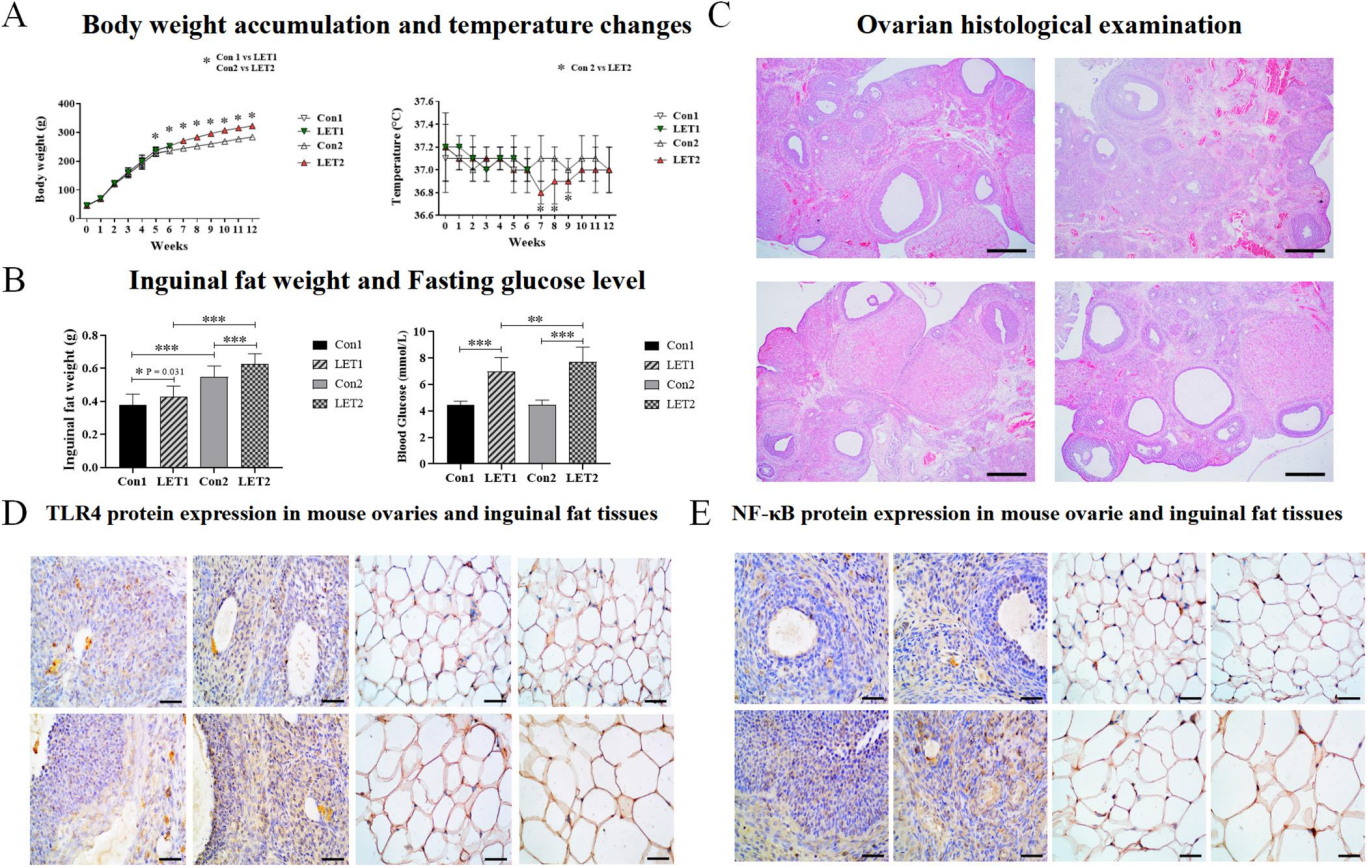
Results of histological analysis.

After implantation of continuous‐release pellets containing either placebo or LET at 21 days of age, body weight accumulation over the following 6 and 12 weeks was as shown in Figure 3A. Significantly increased body weight was observed in rats at 5 and 6 weeks after LET implantation (Con1 vs LET1, *p* < 0.05). In addition, the body weight of the LET2 group was significantly higher than that of the Con2 group during weeks 7–12 (*p* < 0.05). Body temperature fluctuated in the Con1 and Con2 groups, and the body temperature of the LET2 rats was lower than that of the Con2 group in weeks 7, 8, and 9 (Con2 vs LET2, *p* < 0.05; Figure 3A).

#### 3.2.2 Inguinal Fat Weight and Fasting Blood Glucose Level

The weight of inguinal fat tissue of the four groups was compared (Figure 3B). the weight of inguinal fat is increased as the treatment going (Con1 vs Con2, *P* < 0.05). In addition, the weight of inguinal fat in rats at 6 and 12 weeks of LET implantation was higher than control group at the same period (Con1 vs LET1, *p* < 0.05; Con2 vs LET2, *p* < 0.001), and the inguinal fat weight at 12 weeks of LET implantation was higher than that at 6 weeks (*p* < 0.001). This showed that the pathological state of PCOS might be responsible for increased inguinal fat tissue weight. As the severity of PCOS increased, inguinal fat tissue also gradually got weight.

Figure 3B showed the fasting blood glucose level of the four groups. The fasting blood glucose level of rats at 6 and 12 weeks of LET implantation was higher than control group at the same period (Con1 vs LET1, *p* < 0.001; Con2 vs LET2, *p* < 0.001). And the fasting blood glucose level at 12 weeks of LET implantation was higher than that at 6 weeks (*p* < 0.01). This trend suggested that the risk of glucose metabolism disorders was significantly increased in PCOS and became more severe as PCOS disease progresses.

#### 3.2.3 Ovarian Histological Changes

Light microscopy showed that there were more follicles in the ovarian cortex of Con1, Con2, and LET1 groups (Figure 3C), which were in different stages of development. Corpora lutea were clearly visible, cumulus and oocytes were visible, the granulosa cell layer was typically 8–9 cells thick, closely arranged, and mature follicles were intact without hyperemia or edema. The ovaries of the LET2 group had cystic changes, with obvious cystic dilatation of follicles, more atresia, reduced number of granulosa cell layer and luteal body (Figure 3C).

#### 3.2.4 Protein Expression of TLR4 and NF‐κB in Tissues and PCR Array Analysis of NF‐κB Signaling Pathway Target Genes in Ovaries

The abundance of TLR4 and NF‐κB protein expression in ovaries and inguinal fat tissues were measured. We found that the intensity of TLR4 and NF‐κB immunostaining were increased both in ovaries and inguinal fat tissues in LET2(Figure 3D,E).

**Figure 4:**
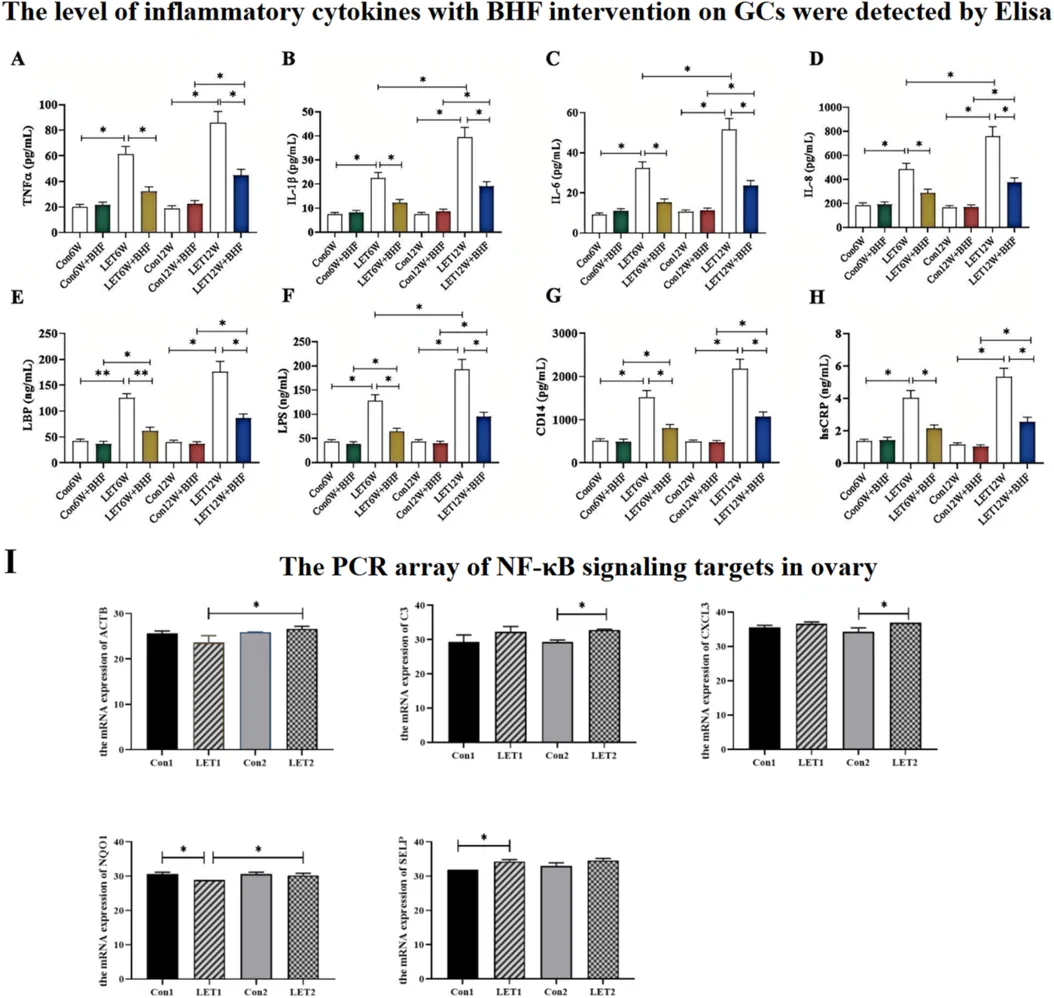
Figure 4A‐4H. The level of inflammatory cytokines with BHF intervention on GCs were detected by Elisa. (Note: These cytokines were included TNFα (A), IL‐1β (B), IL‐6 (C), IL‐8 (D), LBP (E), LPS (F), CD14 (G) and hsCRP(H). *p < 0.05 and **p < 0.01.) Figure 4‐I. The PCR array of NF‐κB signaling targets in ovary. 92 related gene expression profiles were simultaneously analyzed using rat NF‐κB Signaling Targets qPCR Array and there were significant differences in the expression of ACTB (A), C3 (B), CXCL3 (C) NQO1 (D) and SELP (D) genes between groups. Data are mean ± S.E.M.; *p < 0.05.

The genes involved in the NF‐κB signaling was investigated using qRT‐PCR, the results of which were shown in Supporting Information Table S2. There were significant differences in the expressions of ACTB, C3, CXCL3, NQO1, and SELP genes between groups. ACTB expression in LET2 was higher than that in LET1 (*p* < 0.05), shown in Figure 4I. The expression of C3 and CXCL3 in LET2 (Figure 4I)was higher than that in Con2 (*p* < 0.05). The expression of NQO1 in LET1 was higher than that in Con1 and LET2 (*p* < 0.05), shown in Figure 4I. The expression of SELP in LET1 was higher than that in Con1 *(p* < 0.05), shown in Figure 4I.

#### 3.2.5 Elevated Expression of Inflammatory Cytokines in Granulosa Cells of PCOS Rats

The study found that compared with Con 1 and Con 2 groups, the levels of TNF‐α, IL‐1β, IL‐6, IL‐8, LBP, LPS, CD14, and hsCRP were significantly elevated in LET 1 and LET 2 groups (*p* < 0.05), indicating enhanced inflammatory responses in granulosa cells of PCOS rats (Table 5, Figure 4A–H). After 12 weeks of LET administration, the expression levels of IL‐1β, IL‐6, IL‐8, and LPS in granulosa cells were significantly higher than at 6 weeks (*p* < 0.05). TNF‐α, LBP, CD14, and hsCRP levels were also elevated compared to 6 weeks, but the differences were not statistically significant. This suggests that prolonged LET treatment predominantly increases inflammatory responses represented by IL‐1β, IL‐6, IL‐8, and LPS.

**Table 5:** The level of inflammatory cytokines with BHF intervention on GCs of PCOS rats.

Inflammatory factor	Con6W + SC	Con6W + BHF	LET6W + SC	LET6W + BHF	Con12W + SC	Con12W + BHF	LET12W + SC	LET12W + BHF
TNF‐α (pg/mL)	19.74 ± 2.18	21.52 ± 2.35	61.14 ± 6.14^a^	31.95 ± 3.70^c^	18.90 ± 2.23	22.52 ± 2.49	85.83 ± 8.90^a^	44.70 ± 4.73^f,g^
IL‐1β (pg/mL)	7.41 ± 0.78	8.28 ± 0.86	22.49 ± 2.37^a^	12.23 ± 1.37^c^	7.41 ± 0.85	8.72 ± 0.91	39.44 ± 4.11^a,h^	19.10 ± 1.96^f,g^
IL‐6 (pg/mL)	9.02 ± 0.97	10.99 ± 1.11	32.23 ± 3.28^a^	15.29 ± 1.59^a^	10.45 ± 1.09	11.05 ± 1.27	51.55 ± 5.48^a,h^	23.69 ± 2.51^f,g^
IL‐8 (pg/mL)	183.25 ± 19.32	192.85 ± 20.75	484.82 ± 49.09^a^	287.79 ± 31.44^c,e^	165.42 ± 16.50	169.67 ± 18.87	756.90 ± 80.38^a,h^	372.79 ± 41.28^f,g^
LBP (ng/mL)	41.50 ± 4.18	37.03 ± 4.03	125.32 ± 8.27^c^	62.01 ± 6.91^d,e^	39.23 ± 4.5	36.87 ± 3.94	175.69 ± 20.01^a^	86.06 ± 8.21^f,g^
LPS (ng/mL)	42.84 ± 4.72	38.96 ± 4.23	127.39 ± 13.53^a^	64.53 ± 6.96^c,e^	43.35 ± 4.69	39.90 ± 4.14	193.11 ± 20.99^a,h^	94.94 ± 9.79^f,g^
CD14 (μg/mL)	506.96 ± 52.77	492.40 ± 55.37	1511.96 ± 161.32^a^	807.39 ± 85.04^c^	483.36 ± 47.04	472.56 ± 48.82	2172.88 ± 231.71^a^	1072.95 ± 111.55^f,g^

*Note:* a, *p* (Con6W+SC vs. LET6W + SC; Con12W+SC vs. LET12W + SC) < 0.05; b, *p* (Con6W+SC vs. LET6W + SC) < 0.01; c, *p* (LET6W + SC vs. LET6W + BHF) < 0.05; d, *p* (LET6W + SC vs. LET6W + BHF) < 0.01; e, *p* (Con6W +BHF vs. LET6W + BHF < 0.05; f, *p* (LET12W + SC vs. LET12W + BHF) < 0.05; g, *p* (Con12W +BHF vs. LET12W + BHF) < 0.05; h, *p* (LET6W + SC vs. LET12W + SC) < 0.05.

#### 3.2.6 BHF Reduces Inflammatory Cytokine Expression in Granulosa Cells of Pcos Rats After 6 Weeks of LET Intervention

To investigate the effect of BHF on the inflammatory microenvironment of granulosa cells in PCOS rats after 6 weeks of LET administration, pre‐prepared control serum and BHF‐containing serum were co‐cultured with granulosa cells isolated from Con 1 and LET 1 groups for 48 h. The groups were designated as Con6 1, Con 1 + BHF, LET 1, and LET 1 + BHF. Results showed that compared with Con 1, BHF had no significant effect on inflammatory cytokine levels in the Con 1 + BHF group; however, compared with LET 1, BHF reduced the expression of TNF‐α, IL‐1β, IL‐6, IL‐8, LBP, LPS, CD14, and hsCRP in the LET6W + BHF group (*p* < 0.05). This indicates that BHF can reverse the heightened inflammatory state in granulosa cells induced by 6‐week LET implantation in the PCOS rat model (Table 5, Figure 4A–H).

#### 3.2.7 BHF Reduces Inflammatory Cytokine Expression in Granulosa Cells of PCOS Rats After 12 Weeks of LET Intervention

We also examined the effect of BHF on the inflammatory microenvironment of granulosa cells in PCOS rats after 12 weeks of LET administration. Results showed that compared with LET 2, BHF reduced the expression of TNF‐α, IL‐1β, IL‐6, IL‐8, LBP, LPS, CD14, and hsCRP in the LET 2 + BHF group (*p* < 0.05). However, compared with Con 2 + BHF, the LET 2 + BHF group exhibited higher levels of TNF‐α, IL‐1β, IL‐6, IL‐8, LPS, CD14, and hsCRP (*p* < 0.05) as well as LBP (*p* < 0.01). This indicates that while BHF can attenuate the heightened inflammatory response of granulosa cells after 12 weeks of LET administration, it only partially reverses this high‐inflammatory state (Table 5, Figure 4A–H).

## 4 Discussion

In recent years, research on the pathogenesis of PCOS has evolved from conceptualizing it as a purely endocrine and metabolic disorder to recognizing it as a complex pathological process involving CLGI. This study integrated network pharmacology prediction, molecular docking, and experimental validation to systematically investigate the dynamic changes in the early‐stage inflammatory microenvironment of polycystic ovary syndrome (PCOS) and the underlying mechanisms of BHF intervention in the PCOS inflammatory microenvironment. Network pharmacology analysis indicated that 10 active constituents of BHF (eg, luteolin, tanshinone IIA) act on multiple core PCOS targets (AKT1, TNF, IL6, etc). Notably, KEGG pathway enrichment analysis showed that these targets are significantly enriched in NF‐κB signaling and insulin resistance pathways, suggesting that BHF may exert therapeutic effects by regulating these key pathways. Experimental validation demonstrated that BHF significantly downregulated pro‐inflammatory factors (TNF‐α, IL‐1β, IL‐6, IL‐8) in granulosa cells of PCOS animal models, with early intervention (6 weeks) showing superior efficacy compared to mid‐term intervention (12 weeks), underscoring the dynamic progression of PCOS and emphasizing the importance of early treatment.

Although KEGG analysis highlighted several enriched pathways—including the IL‐17, MAPK, insulin resistance, and estrogen signaling pathways—experimental validation was focused on the TLR4/NF‐κB axis for three reasons. First, NF‐κB is the central transcriptional hub of chronic low‐grade inflammation, which is the primary focus of this study. Second, several co‐enriched pathways, such as IL‐17 and MAPK signaling, converge on or cross‐talk with NF‐κB, and the core targets and cytokines identified here (e.g., TNF, IL6, IL1B, and the LPS–LBP–CD14 module) are canonical components or downstream effectors of the TLR4/NF‐κB axis. Third, the insulin resistance and estrogen signaling pathways relate mainly to the metabolic and endocrine features of PCOS, which were beyond the inflammatory scope of the present work. We acknowledge, however, that the other enriched pathways were not examined experimentally, and that the network pharmacology predictions were therefore only partially validated; their roles warrant dedicated investigation in future studies.

Accumulating evidence indicates that PCOS is associated with a chronic low‐grade inflammatory environment mediated by NF‐κB [19]. Aberrant activation of the NF‐κB signaling pathway is closely linked to the production of inflammatory cytokines and serves as a key regulatory hub for ovarian local inflammation in PCOS. TLR4, a member of the Toll‐like receptor family, initiates innate immune responses, modulates adaptive immunity, and defends against pathogens. NF‐κB, a crucial nuclear transcription factor, serves as a key downstream signaling molecule in the TLR4 pathway and activates the transcription of genes involved in inflammatory and immune responses [20]. Lipopolysaccharide (LPS) binds to lipopolysaccharide‐binding protein (LBP) to form a complex, which then interacts with CD14/TLR4 receptors, significantly activating the NF‐κB pathway [21] and elevating downstream pro‐inflammatory factor TNF‐α [22]. TNF‐α, as a core inflammatory mediator, not only further activates NF‐κB via positive feedback but also induces the expression of IL‐6 and IL‐8 [18]. IL‐6 maintains chronic inflammation via the JAK/STAT3 pathway [23]. IL‐8 promotes neutrophil infiltration, exacerbating local ovarian inflammation [24]. Additionally, NF‐κB activation facilitates NLRP3 inflammasome assembly, increasing IL‐1β maturation and release [25]. Clinical studies show that high‐sensitivity C‐reactive protein (hsCRP) levels in PCOS patients positively correlate with NF‐κB activity, suggesting hsCRP as a sensitive marker of NF‐κB pathway activation [26]. Notably, the LPS–NF‐κB axis and these inflammatory factors collectively constitute a regulatory network for chronic low‐grade inflammation in PCOS [27], where NF‐κB both regulates cytokine production and is modulated via positive feedback from the cytokines, forming a persistent inflammatory loop.

Ovarian granulosa cells (GCs) play a critical role in follicular development and the maintenance of normal ovulatory function [28], and their dysfunction constitutes a key pathological basis for anovulation in PCOS. This study observed that GCs from PCOS model rats exhibited significantly elevated levels of TNF‐α, IL‐1β, IL‐6, IL‐8, LBP, LPS, CD14, and hsCRP, leading to activation of the ovarian local NF‐κB inflammatory pathway, thereby establishing a pro‐inflammatory microenvironment in the PCOS ovary. These inflammatory factors interfere with steroidogenesis in GCs, disrupting estrogen functional pathways and ultimately causing anovulation and hormonal abnormalities [23]. BHF treatment reduced inflammatory cytokine levels in GCs of PCOS model rats, ameliorated NF‐κB pathway activation, which may contribute to improvement of the ovarian inflammatory microenvironment in PCOS.

Modern pharmacological studies have shown that BHF contains several well‐characterized active constituents, including astragalus polysaccharides, icariin and atractylenolide [29]. In the present work, network analysis indicated that BHF acts simultaneously on multiple groups of targets involved in inflammation (e.g., TNF and IL‐17), metabolism (e.g., PPARγ and IRS1) and hormone synthesis (e.g., CYP19A1 and HSD3B2). Our experiments verified that BHF reduces eight inflammatory markers (TNF‐α, IL‐1β, IL‐6, IL‐8, LBP, LPS, CD14, and hsCRP) in the NF‐κB pathway of ovarian granulosa cells, highly consistent with the multi‐pathway enrichment predicted by network pharmacology, suggesting its potential to modulate PCOS‐associated inflammation and metabolic dysregulation. This multi‐target, multi‐pathway mechanism may be key to BHF's therapeutic effects in PCOS.

This study validated the dynamic progression characteristics of the inflammatory state in PCOS. Comparative analysis of inflammatory responses and metabolic profiles in PCOS rats following 6‐week and 12‐week LET interventions revealed that the inflammatory state pre‐existed prior to the full establishment of PCOS, concurrently with manifestations of obesity, inguinal fat accumulation, blood glucose abnormalities, and ovarian morphological alterations. Furthermore, the ovarian histopathological changes observed in this study indicated that a substantial number of cystic follicles developed exclusively in the LET 12‐week group. Moreover, the absence of body temperature pulsatility during the 7–8 week and 10–12 week LET intervention periods in the LET 12‐week PCOS rats collectively suggests impaired ovulation function, which may be associated with the pro‐inflammatory microenvironment within the ovaries. Concurrently, time‐course analysis revealed that with prolonged letrozole intervention up to 12 weeks, the levels of inflammatory factors in GCs from model rats were further elevated, indicating the dynamic progression of the inflammatory state in PCOS. Collectively, these findings suggest that a pro‐inflammatory state may be a significant contributing factor to the development of PCOS, and that early identification and intervention targeting the inflammatory microenvironment of GCs could be pivotal in mitigating PCOS progression. These results imply that the pro‐inflammatory ovarian microenvironment in PCOS exhibits a distinct time‐dependent exacerbation pattern, for which early BHF intervention demonstrates more pronounced efficacy. This aligns well with the traditional Chinese medicine concept of “preventing disease progression”, a cornerstone of its preventive medicine philosophy.

It should also be emphasized that these findings were obtained entirely in rat and cell models, and their translation to clinical PCOS requires caution. As a multi‐component, multi‐target herbal formulation, BHF may differ between rats and humans in absorption, metabolism, and effective dose, and the letrozole‐induced model reproduces only part of the heterogeneous phenotype of human PCOS. In particular, the inflammatory changes and the proposed TLR4/NF‐κB mechanism were not validated in human granulosa cells or follicular fluid. Validation in human granulosa cells and follicular fluid, together with clinical cohort studies, will therefore be essential to confirm the relevance of these findings to patients. More broadly, the accurate recognition of endocrine and metabolic disorders often depends on careful mechanistic evaluation, as illustrated even in rarer conditions such as Gitelman syndrome [30]; a mechanism‐oriented understanding of the inflammatory microenvironment, as pursued here, may similarly aid the assessment of women with chronic metabolic conditions such as PCOS.

Several limitations should be acknowledged. First, the specific batch of BHF was not chemically fingerprinted by HPLC or LC‐MS, and its consistency relied on standardized preparation and our previous metabolomic data [13]. Second, the network pharmacology and molecular docking results were computational predictions; additional centrality measures were not included, and these in silico analyzes served only to corroborate the predicted associations rather than to provide definitive validation. Third, only female Wistar rats were studied, and the group size was not based on a formal power analysis. Inflammation was assessed at only two time points (6 and 12 weeks), and reproductive‐endocrine endpoints—serum testosterone, LH, FSH, estradiol, estrous cyclicity, and ovarian steroidogenic genes—were not measured. Consequently, only the trends and features of the inflammatory response could be characterized, and a single strain with this limited set of endpoints may not fully reflect the heterogeneity of human PCOS. Fourth, TLR4/NF‐κB activation was assessed only by immunohistochemistry and a qPCR array of NF‐κB target genes; phosphorylated NF‐κB, IκBα degradation, nuclear translocation, and inhibitor‐ or knockdown‐based experiments were not performed, so the data indicate association rather than causation. Fifth, data were presented as mean ± SEM, without formal correction for multiple comparisons or confidence intervals. Sixth, in the granulosa cell experiments, cell purity and post‐treatment viability were not formally verified, and the 15% concentration of BHF‐containing serum was based on preliminary data rather than a dose–response analysis. Future studies incorporating phytochemical characterization, hormonal and reproductive endpoints, pathway‐specific manipulation, and human validation are therefore warranted.

## 5 Conclusion

This study focuses on elucidating the association between inflammatory status and PCOS characteristics at two distinct time points. During the development of LET‐induced PCOS in rats, the TLR4/NF‐κB pathway remained activated in both inguinal adipose and ovarian tissues. This sustained pro‐inflammatory state may induce or exacerbate the phenotypic features of PCOS. Furthermore, this study explored, using an integrated “network and experimental” strategy, the potential mechanism by which BHF may improve the early low‐grade inflammatory environment in ovarian granulosa cells of PCOS in association with regulation of the NF‐κB pathway. The study demonstrated that granulosa cells of PCOS model rats exhibit significant local inflammation characterized by abnormal activation of multiple inflammatory factors (TNF‐α, IL‐1β, IL‐6, IL‐8, hsCRP, LPS, LBP, CD14) and key signaling pathways (such as NF‐κB).

BHF contains multiple active components capable of significantly inhibiting the expression of these inflammatory factors, downregulating NF‐κB pathway‐related inflammatory factors, and improving the ovarian local inflammatory microenvironment. This suggests its potential to synergistically regulate related proteins such as AKT1, TNF, and IL6, potentially intervening in the early inflammatory processes of PCOS. Furthermore, the high consistency among network pharmacology predictions, molecular docking results, and experimental data provides robust systematic and molecular‐level support for the mechanistic understanding of BHF.

By combining network pharmacology with experimental validation from an integrated traditional Chinese and Western medicine perspective, this study provides preliminary molecular evidence for the TCM concepts of “nourishing the kidney, resolving phlegm, and preventing disease,” and offers experimental support for the potential clinical application of BHF in PCOS. Furthermore, the integration of traditional Chinese and Western medicine, aimed at ameliorating the inflammatory state, may contribute to the prevention and management of PCOS. The modulation of the TLR4/NF‐κB‐associated inflammatory pathway by Chinese herbal compound formulas may present a novel direction for PCOS intervention. Building on these findings, several directions merit further investigation. The inflammatory changes observed here should be validated in human granulosa cells, follicular fluid, and clinical cohorts, and the causal role of the TLR4/NF‐κB axis should be confirmed through inhibitor‐ or knockdown‐based experiments. Incorporating hormonal and reproductive endpoints, together with phytochemical characterization of BHF, would help to define its active constituents and mechanism of action more precisely. Beyond the NF‐κB pathway, the other enriched pathways and different PCOS phenotypes, such as insulin‐resistant and non‐obese subtypes, also deserve attention, so as to advance the individualized and targeted application of traditional Chinese medicine in the treatment of PCOS.

## Author Contributions

Jianing Zhang, Miao Sun and Fangyun Gu conceived and designed the study; Miao Sun and Yunyun Li developed the methodology; Jianing Zhang, Yunyun Li and Fangyun Gu performed the animal experiments, cell culture, ELISA, immunohistochemistry, network pharmacology and molecular docking analyzes; Yunyun Li and Jianing Zhang carried out the formal data analysis and visualization; Fang Xu and Xiaoling Feng provided resources; Jianing Zhang, Miao Sun and Yunyun Li drafted the original manuscript, which was critically reviewed and revised by Miao Sun, Hongying Kuang and Xiaoling Feng; Miao Sun supervised the project and, together with Xiaoling Feng and Hongying Kuang, acquired the funding. Jianing Zhang, Fangyun Gu and Yunyun Li contributed equally to this work and share first authorship. All authors have read and approved the final version of the manuscript, and agree to be accountable for all aspects of the work.

## Conflicts of Interest

The authors declare that the research was conducted in the absence of any commercial or financial relationships that could be construed as a potential conflict of interest.

## Supporting information


Supporting File

## Data Availability

The data that support the findings of this study are available in TCMSP at https://www.tcmsp-e.com/index.php. These data were derived from the following resources available in the public domain: TCMSP, https://www.tcmsp-e.com/index.php. The datasets generated and/or analyzed during the present study are available from the first author (Ms. Jianing Zhang, 1103209053@qq.com) upon reasonable request. Publicly accessible databases used in this work include TCMSP (https://www.tcmsp-e.com/), UniProt (https://www.uniprot.org/), GeneCards (https://www.genecards.org/), OMIM (http://www.omim.org/), STRING (https://www.string-db.org/), Metascape (https://metascape.org/), and the RCSB Protein Data Bank (https://www.rcsb.org/). No large‐scale sequencing or proteomics datasets were generated, and therefore no accession number has been deposited in any public repository.
