# Supplementary material for: Bushen Huatan Formula Ameliorates Early Chronic Low‐Grade Inflammation in Obese PCOS Via the TLR4/NF‐κB Signaling Pathway: An Integrated Network Pharmacology and Experimental Study
Source: Immun Inflamm Dis. 2026 Sep 14;14(9):e70518. doi: 10.1002/iid3.70518 (PMC13573318; doi:10.1002/iid3.70518)
Supplement: Supplementary file 1 [file IID3-14-e70518-s001.docx]

Supplementary TableS1: Genes of NF-κB signal target array.

| rat | 1 | 2 | 3 | 4 | 5 | 6 |
| --- | --- | --- | --- | --- | --- | --- |
| A | ACTB | CCR5 | F3 | IL2RA | NLRP12 | TLR1 |
| B | ADM | CD40 | F8 | IL4 | NQO1 | TLR2 |
| C | AGT | CD74 | FAS | IL6 | NR4A2 | TLR3 |
| D | AKT1 | CD80 | FASLG | INS2 | PDGFB | TLR4 |
| E | ALDH3A2 | CD83 | GADD45B | IRF1 | PLAU | TNF |
| F | BCL2 | CDKN1A | GAPDH | LTA | PTGS2 | TNFRSF1B |
| G | BCL2L1 | CSF1 | HPRT1 | LTB | REL | TNFSF10 |
| H | BCL3 | CSF2 | ICAM1 | MAP2K6 | RELA | TNFSF14 |
| I | BIRC2 | CSF2RB | IFNB1 | MITF | SELE | TP53 |
| J | C3 | CSF3 | IFNG | MMP9 | SELP | TRAF2 |
| K | C4B | CXCL1 | IL12B | MYC | SNAP25 | UBB |
| L | CCL11 | CXCL10 | IL1A | MYD88 | SOD2 | VCAM1 |
| M | CCL12 | CXCL3 | IL1B | NCOA3 | STAT1 | - |
| N | CCL22 | CXCL9 | IL1R2 | NFKB1 | STAT3 | - |
| O | CCL5 | EGFR | IL1RN | NFKB2 | STAT5B | - |
| P | CCND1 | EGR2 | IL2 | NFKBIA | TBP | - |

Supplementary TableS2: The NF-κB signaling targets PCR array in ovary.

| Inflamation  cytokines | Con1 | LET1 | Con2 | LET2 |
| --- | --- | --- | --- | --- |
| **ACTB** | 25.60±0.57 | 23.60±1.53 | 25.93±0.01 | 26.60±0.58^▲^ |
| **ADM** | 30.92±0.01 | 30.92±1.00 | 30.59±0.58 | 31.26±0.57 |
| **AGT** | 30.93±1.01 | 31.92±3.60 | 30.93±1.00 | 32.59±1.53 |
| **AKT1** | 26.57±0.58 | 26.91±1.00 | 26.58±0.57 | 26.91±0.01 |
| **ALDH3A2** | 27.92±0.00 | 28.93±1.00 | 28.25±0.59 | 28.92±0.01 |
| **BCL2** | 30.91±0.01 | 30.58±0.58 | 30.25±0.59 | 30.91±0.01 |
| **BCL2L1** | 30.25±0.58 | 31.25±0.58 | 30.58±0.58 | 30.25±0.58 |
| **BCL3** | 32.28±0.58 | 33.28±1.15 | 33.29±0.58 | 33.62±0.58 |
| **BIRC2** | 31.59±3.19 | 28.63±1.16 | 27.57±0.59 | 28.58±0.59 |
| **C3** | 29.26±2.08 | 32.26±1.53 | 29.26±0.58 | 32.81±0.20^★^ |
| **C4B** | 28.93±0.01 | 29.59±1.52 | 28.93±0.00 | 29.26±0.58 |
| **CCL11** | 30.24±0.58 | 29.57±1.15 | 30.90±0.00 | 29.90±1.00 |
| **CCL12** | 29.26±0.58 | 29.93±1.00 | 29.92±0.00 | 30.59±2.08 |
| **CCL22** | 36.40±0.70 | 35.59±0.59 | 34.40±0.71 | 36.24±0.57 |
| **CCL5** | 27.93±0.00 | 28.59±1.15 | 28.59±0.58 | 29.59±0.58 |
| **CCND1** | 27.92±0.00 | 28.59±0.58 | 27.92±0.00 | 28.26±0.58 |
| **CCR5** | 35.25±0.57 | 36.60±0.59 | 36.26±0.57 | 36.25±0.57 |
| **CD40** | 31.25±0.58 | 31.59±1.15 | 31.58±1.16 | 32.25±0.57 |
| **CD74** | 25.92±0.01 | 26.19±1.92 | 25.92±0.01 | 26.83±0.46 |
| **CD80** | 31.57±0.58 | 31.91±0.01 | 31.90±1.00 | 31.91±1.00 |
| **CD83** | 32.93±0.00 | 32.94±0.01 | 33.27±0.58 | 33.27±0.57 |
| **CDKN1A** | 35.91±1.01 | 35.97±1.10 | 36.25±0.58 | 36.69±0.66 |
| **CSF1** | 28.92±0.01 | 29.59±0.58 | 28.92±0.00 | 29.59±0.58 |
| **CSF2** | 35.26±0.58 | 36.00±1.52 | 36.33±0.70 | 34.92±0.00 |
| **CSF2RB** | 31.59±0.58 | 32.92±1.00 | 31.59±0.58 | 31.93±0.00 |
| **CSF3** | 35.26±0.58 | 35.59±0.57 | 34.59±0.58 | 35.59±1.53 |
| **CXCL1** | 29.59±0.58 | 29.59±0.57 | 30.59±0.58 | 30.26±2.08 |
| **CXCL10** | 31.58±0.58 | 31.92±1.74 | 31.25±0.58 | 32.71±1.06 |
| **CXCL3** | 35.58±0.58 | 36.59±0.57 | 34.25±1.16 | 36.91±0.00^★^ |
| **CXCL9** | 26.92±0.00 | 25.59±1.16 | 26.26±0.58 | 25.92±1.00 |
| **EGFR** | 27.90±0.01 | 28.57±1.52 | 28.57±0.57 | 28.58±0.58 |
| **EGR2** | 32.60±1.16 | 32.67±0.58 | 32.27±1.15 | 32.60±1.15 |
| **F3** | 29.58±0.57 | 29.25±1.53 | 29.92±0.00 | 30.25±0.58 |
| **F8** | 31.93±1.00 | 32.26±0.58 | 31.93±0.00 | 32.60±0.58 |
| **FAS** | 29.57±0.57 | 30.24±2.30 | 29.58±0.58 | 30.24±0.57 |
| **FASLG** | 32.25±0.58 | 31.59±0.57 | 31.59±0.58 | 31.92±1.00 |
| **GADD45B** | 28.27±1.16 | 29.27±0.58 | 28.60±0.58 | 28.93±0.00 |
| **GAPDH** | 22.59±0.58 | 24.25±0.58 | 23.59±1.53 | 24.25±1.16 |
| **HPRT1** | 22.92±0.00 | 23.59±1.16 | 23.59±0.58 | 23.93±0.00 |
| **ICAM1** | 31.26±0.58 | 31.60±0.58 | 31.27±0.58 | 31.27±0.58 |
| **IFNB1** | 33.59±1.16 | 34.59±0.58 | 32.57±0.58 | 33.59±1.15 |
| **IFNG** | 33.90±1.00 | 33.90±1.00 | 33.23±0.59 | 33.23±1.53 |
| **IL12B** | 32.93±1.00 | 31.93±0.00 | 32.60±0.58 | 32.60±1.15 |
| **IL1A** | 32.92±1.00 | 33.92±1.73 | 31.92±1.00 | 32.59±1.15 |
| **IL1B** | 29.26±0.57 | 29.60±1.53 | 28.92±1.00 | 30.60±0.58 |
| **IL1R2** | 36.24±0.60 | 36.64±0.63 | 36.23±0.57 | 36.94±0.05 |
| **IL1RN** | 31.59±0.58 | 30.93±1.00 | 31.92±0.00 | 31.59±0.57 |
| **IL2** | 34.92±1.00 | 34.25±0.58 | 34.41±0.70 | 35.41±0.70 |
| **IL2RA** | 37.08±0.60 | 37.04±0.52 | 37.02±0.77 | 37.09±0.69 |
| **IL4** | 33.26±2.50 | 34.92±1.01 | 33.93±0.98 | 35.64±1.59 |
| **IL6** | 33.26±2.50 | 34.92±1.01 | 33.93±0.98 | 35.64±1.59 |
| **INS2** | 29.93±1.01 | 29.93±0.00 | 29.94±0.00 | 29.93±0.00 |
| **IRF1** | 29.93±1.01 | 29.93±0.00 | 29.94±0.00 | 29.93±0.00 |
| **LTA** | 32.60±0.58 | 32.94±1.74 | 32.27±0.58 | 33.60±0.58 |
| **LTB** | 30.23±0.57 | 30.57±0.58 | 30.56±0.58 | 30.90±0.00 |
| **MAP2K6** | 29.93±1.00 | 29.93±1.00 | 29.93±0.00 | 30.27±0.58 |
| **MITF** | 29.93±1.00 | 29.93±1.00 | 29.93±0.00 | 30.27±0.58 |
| **MMP9** | 33.28±0.58 | 33.27±0.58 | 33.27±0.58 | 34.27±0.58 |
| **MYC** | 29.27±0.58 | 30.27±0.58 | 29.61±0.58 | 29.94±0.01 |
| **MYD88** | 31.26±0.57 | 31.93±1.00 | 31.93±0.00 | 31.60±0.58 |
| **NCOA3** | 28.60±0.58 | 28.93±1.00 | 28.94±0.01 | 28.93±0.00 |
| **NFKB1** | 28.60±0.58 | 28.93±1.00 | 28.94±0.01 | 28.93±0.00 |
| **NFKB2** | 26.56±0.57 | 26.56±0.58 | 26.89±0.00 | 26.57±0.58 |
| **NFKBIA** | 34.94±1.73 | 35.94±1.00 | 34.26±0.58 | 35.27±1.16 |
| **NLRP12** | 29.60±0.58 | 30.60±0.58 | 30.60±0.58 | 30.26±0.58 |
| **NQO1** | 30.59±0.58 | 28.93±0.00^#^ | 30.59±0.58 | 30.26±0.58^▲^ |
| **NR4A2** | 30.26±0.58 | 31.27±1.16 | 30.94±0.00 | 31.27±0.58 |
| **PDGFB** | 30.27±0.58 | 29.94±1.00 | 29.94±0.00 | 30.60±0.58 |
| **PLAU** | 34.25±1.53 | 34.58±1.56 | 34.25±0.58 | 34.58±1.53 |
| **PTGS2** | 35.58±0.58 | 35.26±1.53 | 35.59±0.57 | 35.26±1.52 |
| **REL** | 27.58±0.58 | 27.59±0.57 | 27.91±0.00 | 27.91±0.00 |
| **RELA** | 27.58±0.58 | 27.59±0.57 | 27.91±0.00 | 27.91±0.00 |
| **SELE** | 37.21±0.16 | 37.06±0.08 | 37.07±0.10 | 37.1±0.00 |
| **SELP** | 31.93±0.00 | 34.27±0.58^#^ | 32.93±1.00 | 34.59±0.58 |
| **SNAP25** | 24.95±0.01 | 25.94±1.73 | 25.28±0.58 | 26.61±0.58 |
| **SOD2** | 26.26±0.58 | 26.93±1.00 | 25.93±0.00 | 26.60±0.57 |
| **STAT1** | 25.90±0.99 | 26.91±1.00 | 26.24±0.58 | 27.24±0.57 |
| **STAT3** | 27.93±1.00 | 28.60±0.58 | 27.93±0.00 | 27.93±0.01 |
| **STAT5B** | 27.94±1.00 | 28.60±0.58 | 27.93±0.00 | 27.93±0.01 |
| **TBP** | 29.60±0.58 | 30.26±1.16 | 30.27±0.58 | 30.26±0.58 |
| **TLR1** | 36.27±0.58 | 36.61±0.59 | 35.59±1.53 | 36.93±0.00 |
| **TLR2** | 28.56±0.58 | 28.24±0.58 | 28.24±0.57 | 28.56±0.57 |
| **TLR3** | 29.25±0.57 | 29.58±1.15 | 29.25±0.58 | 29.58±0.58 |
| **TLR4** | 32.25±0.58 | 32.24±0.58 | 32.24±0.57 | 32.58±1.16 |
| **TNF** | 30.92±0.00 | 30.27±0.58 | 30.27±0.57 | 30.60±0.58 |
| **TNFRSF1B** | 28.23±0.58 | 28.90±0.00 | 27.90±0.01 | 28.57±0.58 |
| **TNFSF10** | 28.23±0.58 | 28.90±0.00 | 27.90±0.01 | 28.57±0.58 |
| **TNFSF14** | 26.93±1.00 | 27.60±1.15 | 26.93±0.00 | 27.93±1.00 |
| **TP53** | 31.93±1.01 | 31.94±1.00 | 31.26±0.57 | 31.93±0.00 |
| **TRAF2** | 22.60±0.58 | 22.60±0.58 | 22.93±0.00 | 22.93±0.01 |
| **UBB** | 30.93±0.00 | 31.60±2.08 | 30.93±0.00 | 31.93±1.00 |
| **VCAM1** | 32.61±1.16 | 32.95±1.00 | 32.95±0.00 | 32.95±0.02 |

Note: ^#^: *P* Con1 vs LET1＜0.05; ^▲^: *P* LET1 vs LET2＜0.05; ^★^: *P* Con2 vs LET2＜0.05.

Supplementary TableS3: Study design, group allocation, and downstream assays

| Group | Treatment (continuous-release pellet) | N | Sacrifice | Downstream assays |
| --- | --- | --- | --- | --- |
| Con1 | Placebo  60-day | 20 | 6 weeks | Weekly body weight, food intake, body temperature; fasting blood glucose; inguinal fat weight; ovary & fat H&E and IHC (TLR4, NF-κB); ovarian NF-κB qPCR array; GC isolation → ELISA (8 cytokines) |
| LET1 | Letrozole 200  μg/day, 60-day | 20 | 6 weeks | Weekly body weight, food intake, body temperature; fasting blood glucose; inguinal fat weight; ovary & fat H&E and IHC (TLR4, NF-κB); ovarian NF-κB qPCR array; GC isolation → ELISA (8 cytokines) |
| Con2 | Placebo  90-day | 20 | 12 weeks | Weekly body weight, food intake, body temperature; fasting blood glucose; inguinal fat weight; ovary & fat H&E and IHC (TLR4, NF-κB); ovarian NF-κB qPCR array; GC isolation → ELISA (8 cytokines) |
| LET2 | Letrozole 200  μg/day, 90-day | 20 | 12 weeks | Weekly body weight, food intake, body temperature; fasting blood glucose; inguinal fat weight; ovary & fat H&E and IHC (TLR4, NF-κB); ovarian NF-κB qPCR array; GC isolation → ELISA (8 cytokines) |

（Note：Of the 20 rats per group, 10 were used for histology/immunohistochemistry and 10 for granulosa cell (GC) isolation. For the in vitro arm, GCs from each group were cultured for 48 h and then treated for a further 48 h with 15% BHF-containing serum or 15% control serum; supernatants were assayed by ELISA for TNF-α, IL-1β, IL-6, IL-8, LBP, LPS, CD14, and hsCRP.）
